# Supplementary material for: Neurovascular coupling and CO2 interrogate distinct vascular regulations
Source: Nat Commun. 2024 Sep 2;15:7635. doi: 10.1038/s41467-024-49698-9 (PMC11369082; doi:10.1038/s41467-024-49698-9)
Supplement: Supplementary file 1 — Supplementary Information [file 41467_2024_49698_MOESM1_ESM.docx]

**Supplementary Informations**


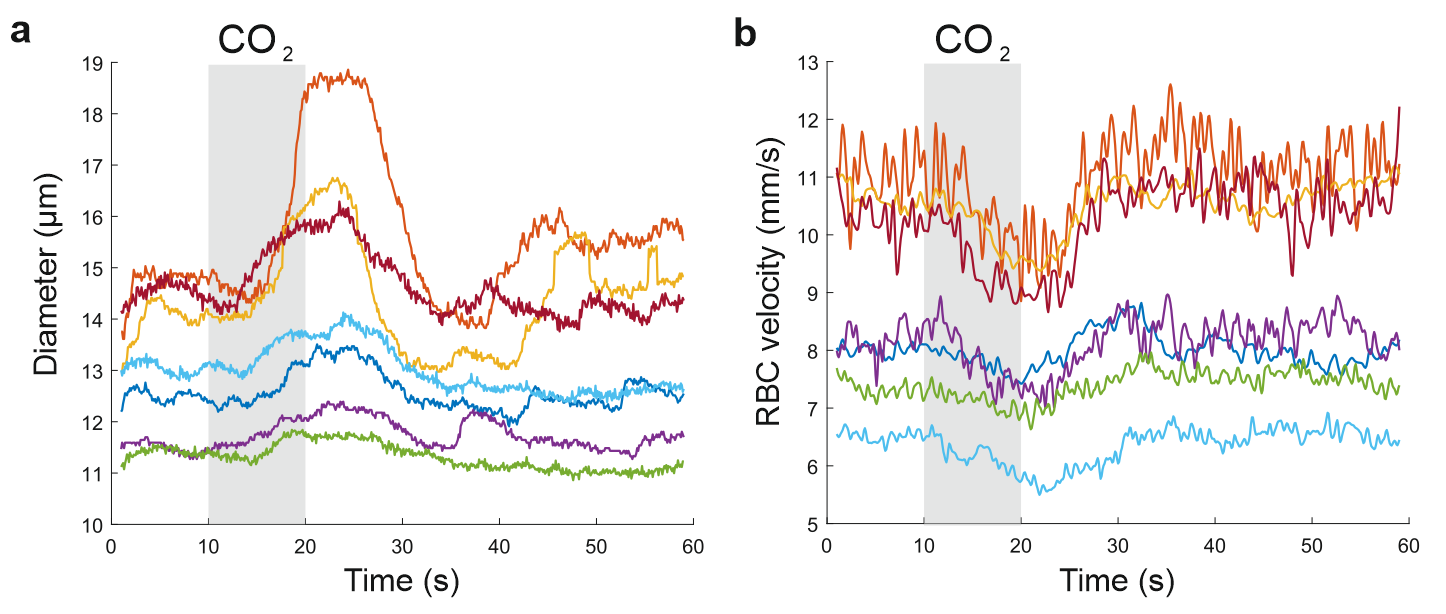


**Supplementary Figure 1. Raw data from Figure 2.**

**a,** Diameter and **b,** red blood cell (RBC) velocity simultaneously measured in pial arterioles using broken line scans to calculate blood flow changes upon briefCO_2_ stimulation. n = 7 vessels, 6 mice.


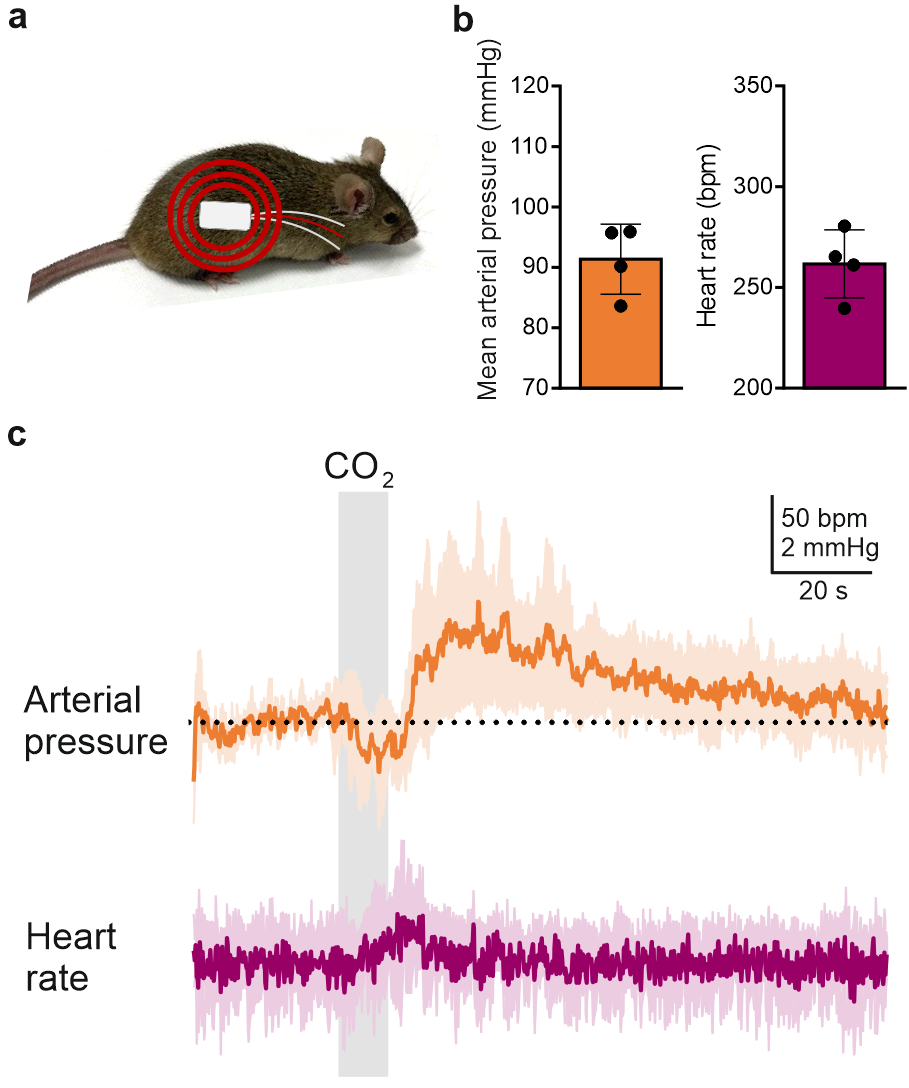


**Supplementary Figure 2. briefCO_2_ causes an early drop in blood pressure.**

**a,** Mice were chronically implanted with a blood pressure telemetric system as described in Methods. **b,** Resting mean arterial pressure and heart rate under dexmedetomidine sedation (n = 4 mice). Data are represented as mean ± SD. Note that dexmedetomidine sedation leads to a marked reduction of heart rate and a mild hypotension (expected values in awake C57/Bl6 mice during the diurnal phase^1,2^: 550 bpm, 100 mmHg). **c,** Top, BriefCO_2_ induces a rapid drop in arterial pressure (orange trace) of about 1 mmHg, lasting the duration of the stimulus (10 s), and followed by a positive rebound. Bottom, BriefCO_2_ induces a reversible increase in heart rate (purple trace, average of 4 to 8 trials, 4 mice). Data are represented as mean ± SD (shadings).


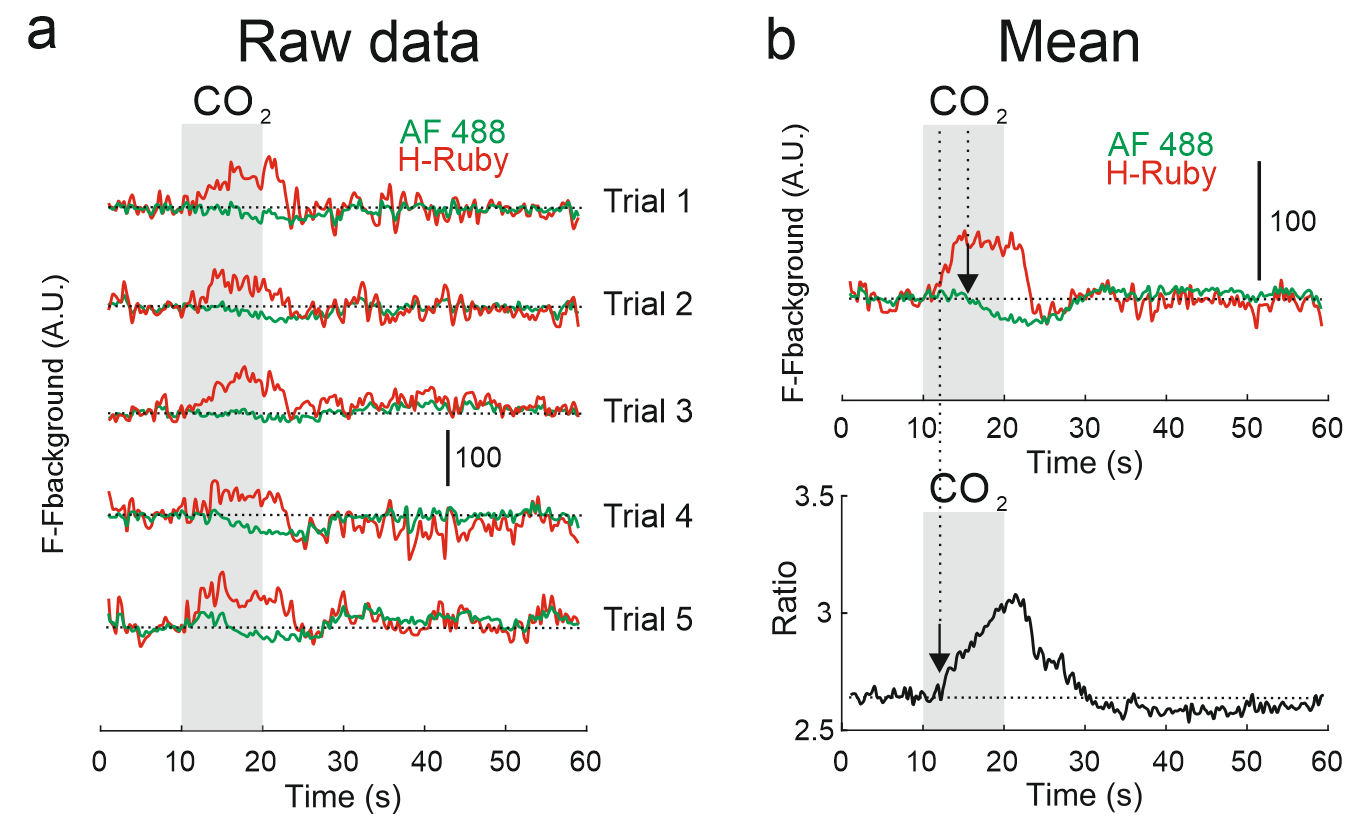


**Supplementary Figure 3. The necessity of dye ratioing.**

**a,** AF 488 (green traces, pH insensitive) and H-Ruby (red traces, pH sensitive) fluorescence changes measured in a pial arteriole in a mouse in response to 5 consecutive briefCO_2_ stimulations. For both dyes, F_background_ was measured in the tissue outside the pial vessel. **b,** Top, averages of the 5 trials. Note the rapid increase of the H-Ruby signal (red trace), indicating the early onset of pial arteriole acidosis, and the delayed drop of AF488 fluorescence (green trace), in phase with the vessel dilation. This shows the importance of dual simultaneous measurements to get rid of the bias due to changes in the excitation beam resulting from changes in optical aberrations induced by the vessel or changes in absorption of excitation light by hemoglobin. Bottom, the fluorescence ratio (black trace) reveals the real shape the pH change.


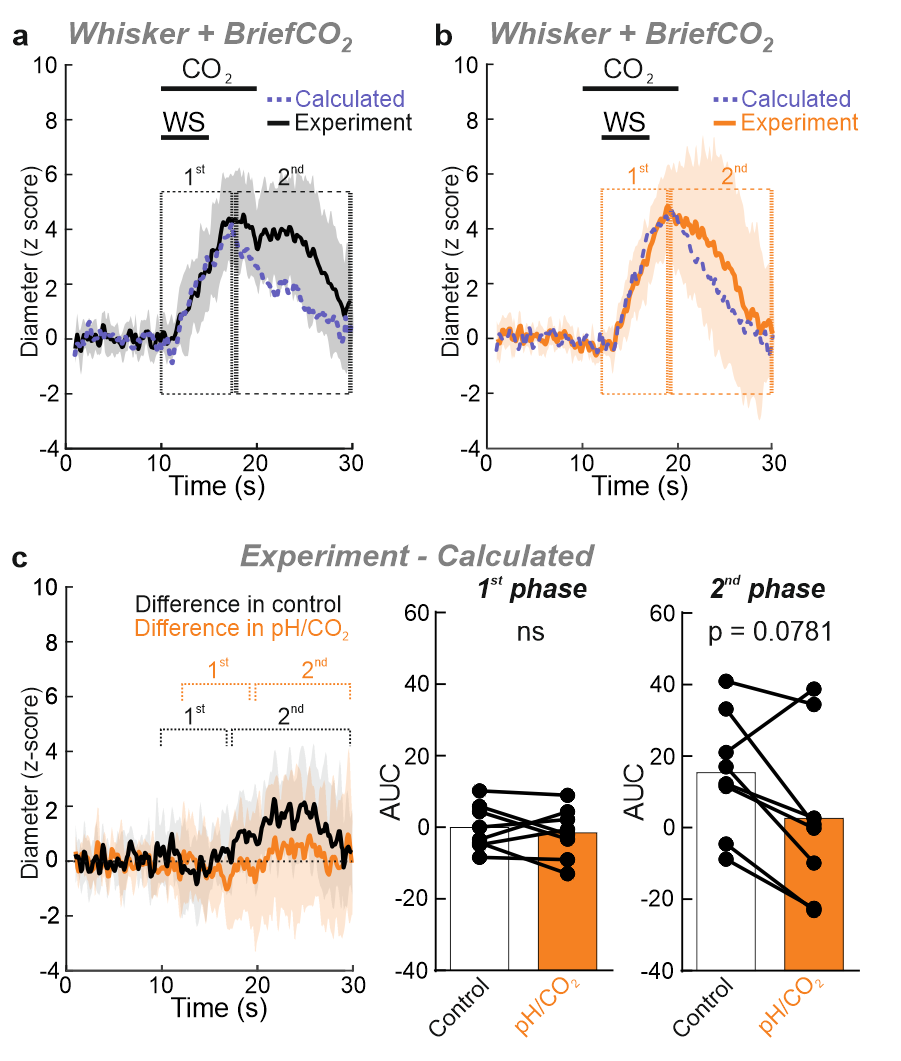


**Supplementary Figure 4. Additivity of NVC and responses to briefCO_2_.**

**a,** Calculated summation (dotted purple line) and experimental data (black solid line) of the responses to whisker stimulation and briefCO_2_ applied at the same time. **b,** Calculated summation (dotted purple line) and experimental data (orange solid line) when whisker stimulation is delayed by 2 s from the onset of briefCO_2_. **c,** Left, Difference between calculated and experimental vascular responses in both conditions. Middle, AUC for area under the curve during the first phase of the response (between 10-17 s and 12-19 s, i.e. when WS is simultaneous or delayed by 2s from briefCO_2_). Paired data, two-sided Wilcoxon sum rank test, ns, p = 0.4609. Right, AUC during the second phase of the response. Paired data, two-sided Wilcoxon sum rank test, ns, p = 0.0781. n = 8 vessels, 5 mice. Data are represented as mean ± SD (shadings).


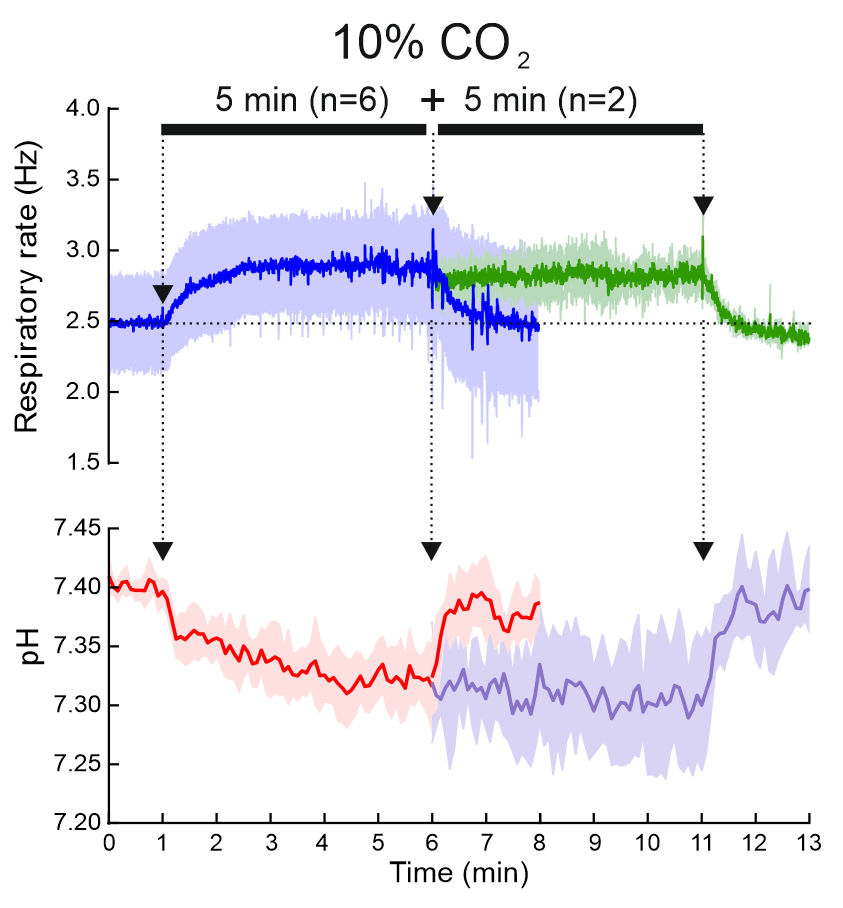


**Supplementary Figure 5. Prolonged CO_2_ stimulation generates a sustained blood acidosis.**

10% CO_2_ stimulation for 5 minutes (1^st^ segments, blue and red traces, n = 6 experiments, 3 mice) or 10 minutes (2^nd^ segments, green and purple traces, n = 2 additional experiments, 2 mice) induces a sustained increase of respiratory rate (top) and a continuous arteriolar acidification (bottom). Data are represented as mean ± SD (shadings).


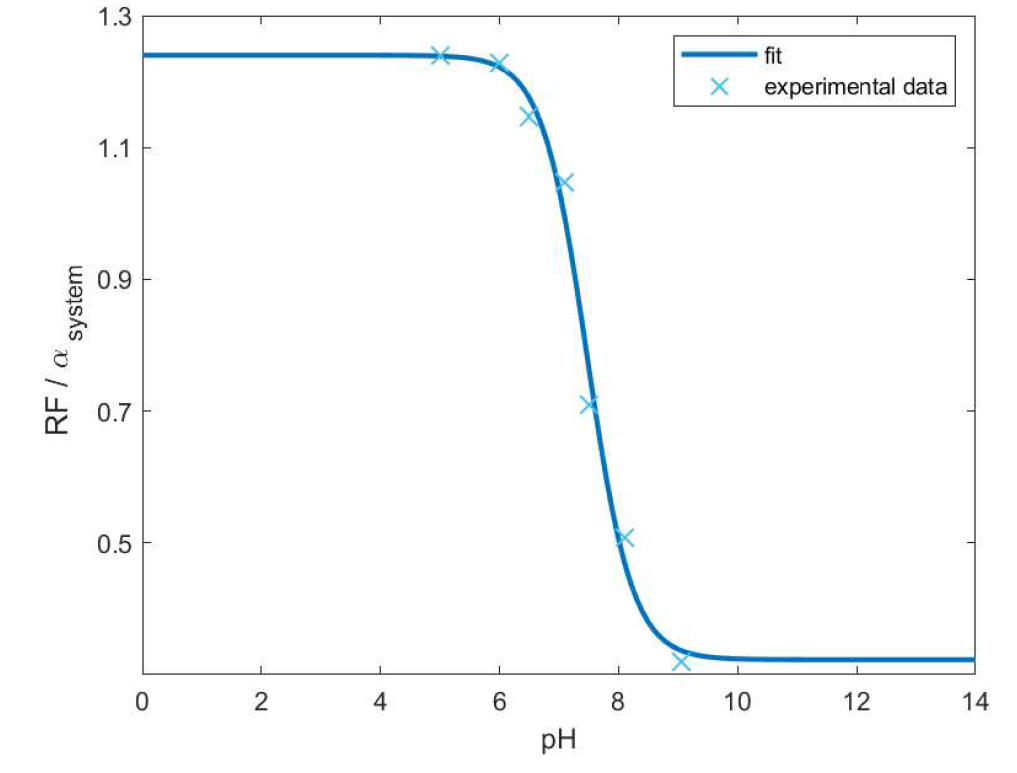


**Supplementary Figure 6. Calibration used for the look-up table.**

A look-up table was established by simultaneously measuring red and green fluorescence of a solution of H-Ruby-dextran and AF488 dextran in fresh rat blood plasma. pH was controlled with a pH sensitive electrode. The ratio of the average red fluorescence over the average green fluorescence (RF) was normalized by the relative sensitivity of the system (α_system_) and plot as a function of pH, fitted with a sigmoid function and used as a reference to calibrate in vivo pH measurements.

| Diameter | 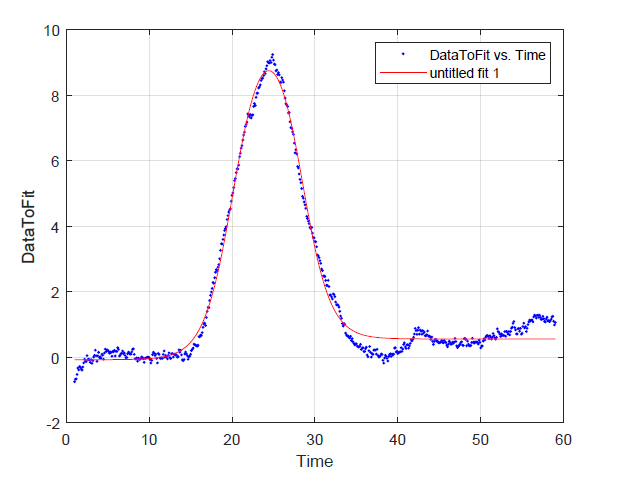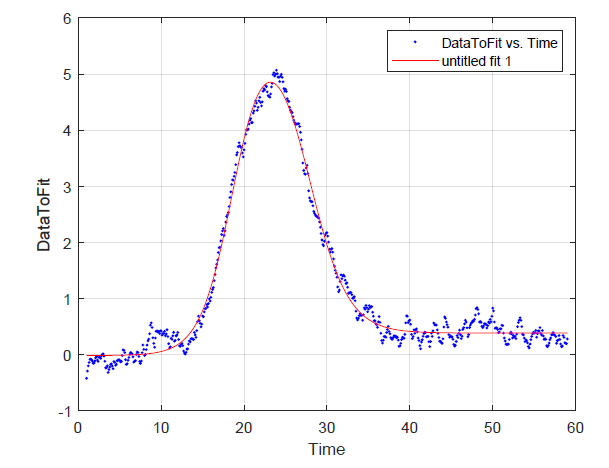  Average diameter responses of pial (left) and penetrating arterioles (right) (z score, data from Figure 1) were fitted with the sum of two sigmoids:  $\frac{a_{0}}{1 + e^{- \frac{\left( x-x_{0} \right)}{T_{0}}}}+ \frac{a_{1}}{1 + e^{\frac{\left( x-x_{1} \right)}{T_{1}}}} + c$ (1). |
| --- | --- |
| Respiratory rate | **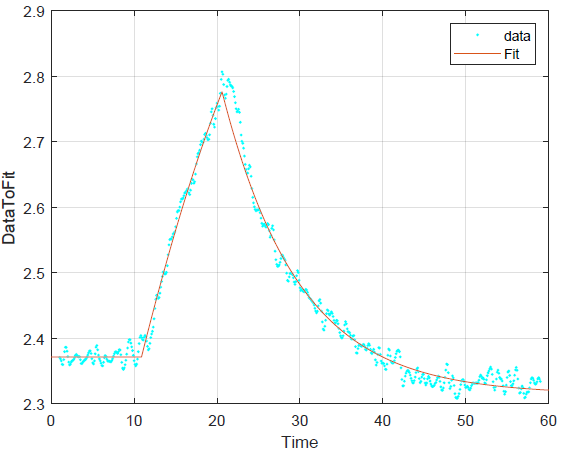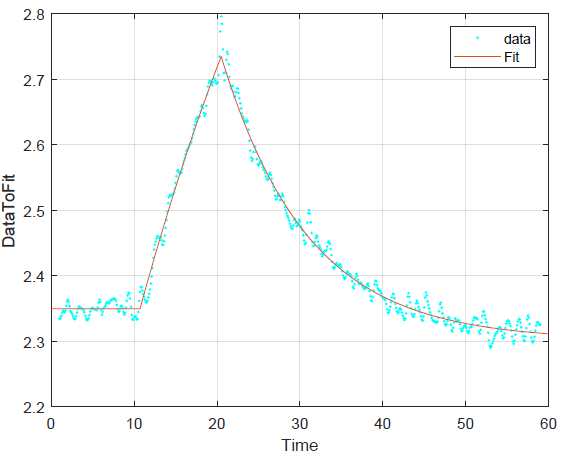**  Average respiratory rate responses (measurements paired with pial (left) and penetrating arterioles (right); data in Hz, data from Figure 1) were fitted with a model function, composite of a steady state baseline, a second-order polynomial for the rising phase:  $a_{1}{(x - T_{1})}^{2}+ b_{1}(x - T_{1})+ c_{1}$ (2),  and the sum of two exponentials for the decay phase:  $a_{2} e^{- \frac{\left( x - x_{2} \right)}{T_{2}}}+ a_{3} e^{- \frac{\left( x - x_{3} \right)}{T_{3}}}+ c_{2}$ (3). |
| RBC velocity (pial arterioles) | 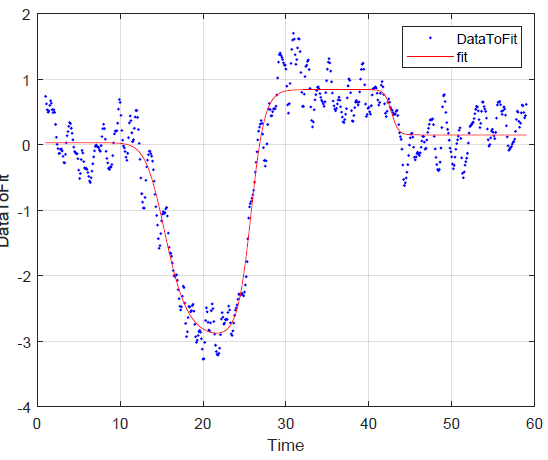  Average velocity responses of pial arterioles (z score, data from Figure 2) were fitted with a sum of two sigmoids and the square of a 3^rd^ sigmoid:  $\frac{a_{0}}{1 + e^{- \frac{\left( x-x_{0} \right)}{T_{0}}}}+ \frac{a_{1}}{1 + e^{\frac{\left( x-x_{1} \right)}{T_{1}}}} +\frac{a_{2}}{\left( 1 + e^{\frac{\left( x-x_{2} \right)}{T_{2}}} \right)^{2}}+ c$ (4). |
| GCaMP6/8 fluorescence | **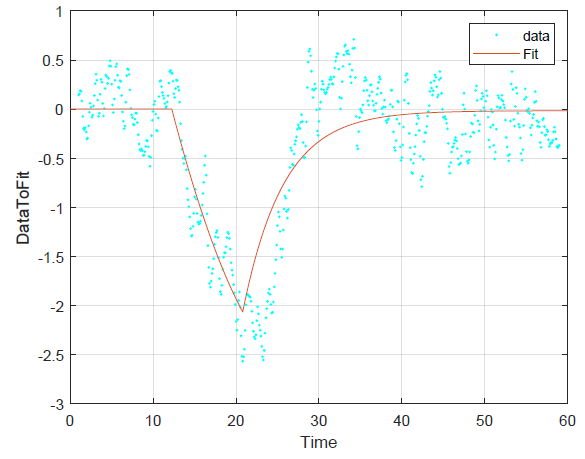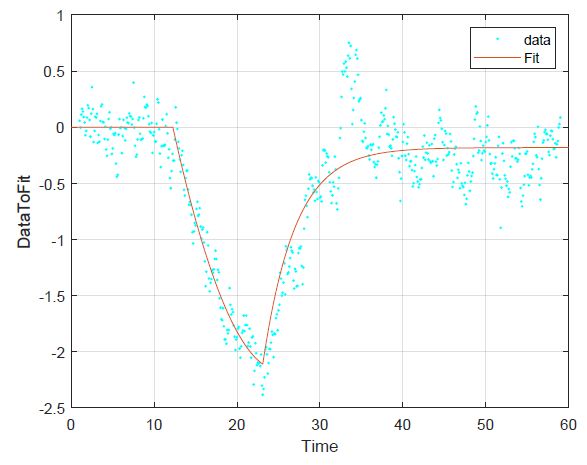**  **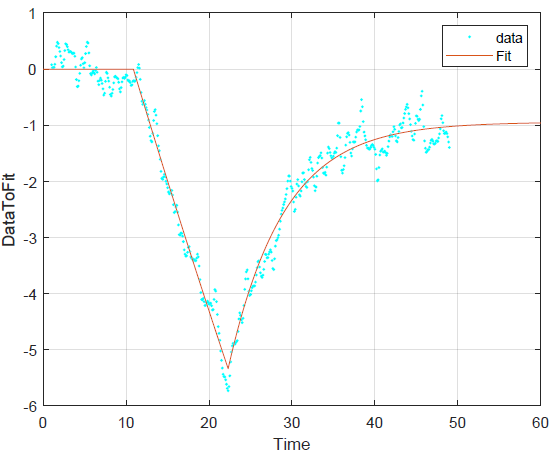 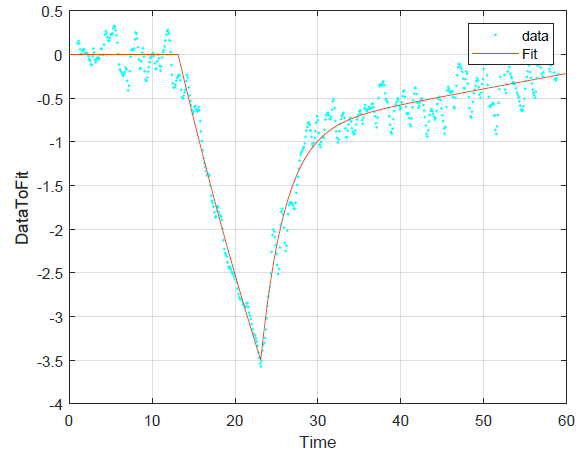**  Average fluorescence responses of smooth muscle cells (top left), endothelial cells (top right), astrocytes (bottom left), and neuropil (bottom right) (z score, data from Figure 3) were fitted with a model function, composite of a steady state baseline, a second-order polynomial for the initial phase of the response as in Eq. (2), and the sum of two exponentials for the late phase as in Eq. (3). |
| CBV (fUS) | 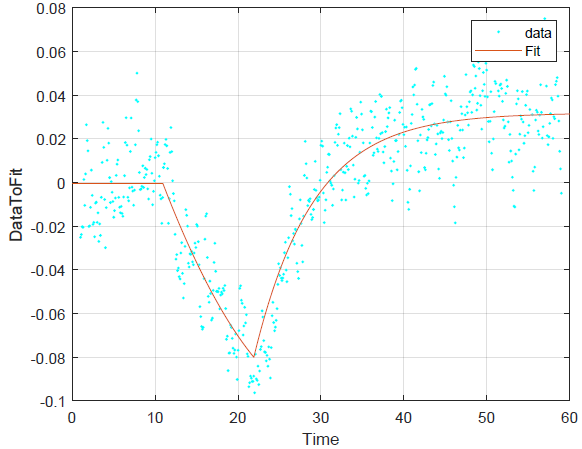 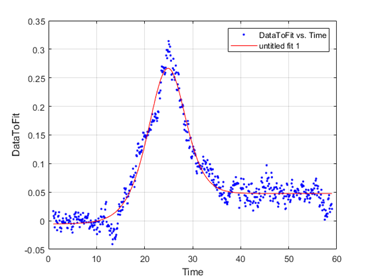  Average CBV responses in the carotid artery (left, z score, data from Figure 2) were fitted with a composite function made of a steady state baseline, a second-order polynomial for the initial phase of the response as in Eq. (2) and an exponential for the late phase:  $a_{2} e^{- \frac{\left( x - x_{2} \right)}{T_{2}}}+ c_{2}$ (5).  Average CBV responses in the cortex (right, z score, data from Figures 2 and 5) were fitted with the sum of two sigmoids as in Eq. (1). |
| Blood flow in arterioles | 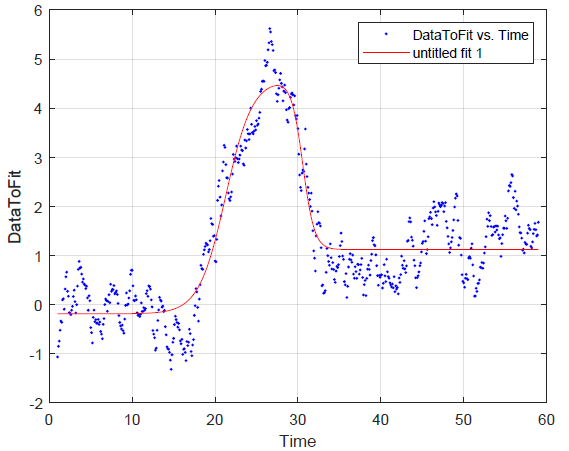  Average blood flow responses in the pial arterioles (z score, data from Figure 2) were fitted with the sum of two sigmoids as in Eq. (1).  Note that the negativity was on purpose not considered for the fit. |
| Intravascular pH | **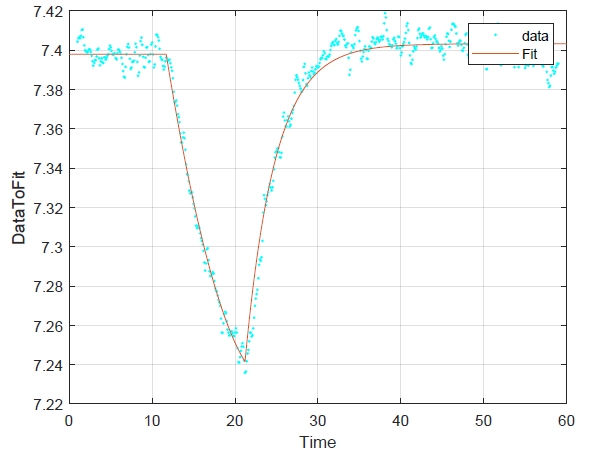**  Average intravascular pH (measured from the ratio of H-Ruby-dextran and AF488-dextran) was fitted with a model function, composite of a steady state baseline, a second-order polynomial for the initial phase of the response as in Eq. (2), and the sum of two exponentials for the late phase as in Eq. (3). |

**Supplementary Table 1. Average responses, fits (in red) and model functions used to compute the fits.**

**Synthesis of H-Ruby Dextran conjugate**

**Synthesis of H-Ruby-N_3_**

**1.** To a solution of 1-Boc-piperazine (1.0 g, 5.4 mmol, 1 eq.) and K_2_CO_3_ (0.89 g, 6.4 mmol, 1.2 eq.) in DCM (10 mL) was added chloroacetyl chloride (470 µL, 5.9 mmol, 1.1 eq.) dropwise at 0 °C. The reaction mixture was stirred for 1 h at room temperature. The mixture was diluted with DCM (30 mL) and washed with water, HCl 1M and brine. The extracted organic layer was dried over anhydrous MgSO_4_, filtered and evaporated to afford the desired product as a yellow solid (1.38 g, 97%). ^1^H NMR is in accordance with the literature^3^.


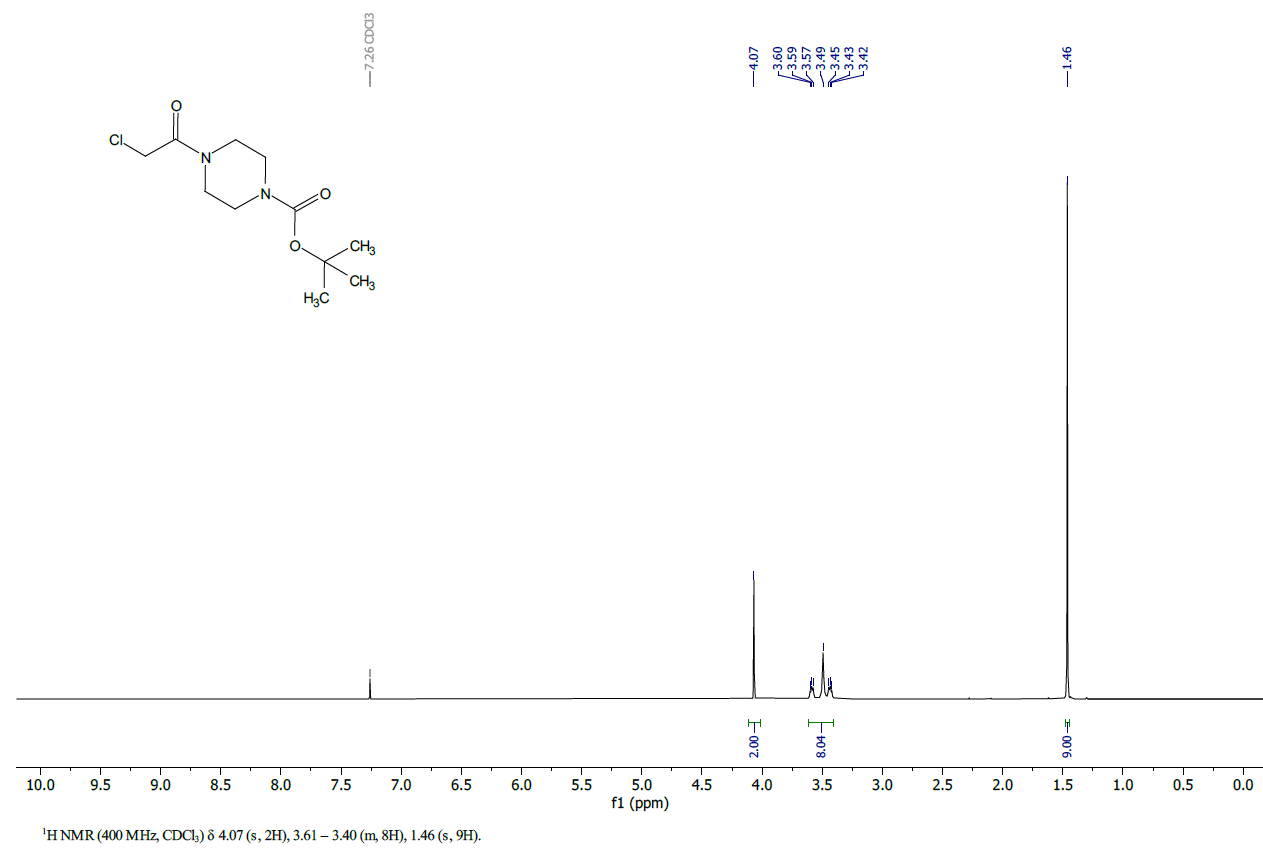


^1^H NMR spectrum of **1**

**2.** Piperazine **1** (1.4 g, 5.5 mmol, 1 eq.) was dissolved in a H_2_O/Acetone mixture (1:1, 20 mL). NaI (0.99 g, 6.6 mmol, 1.2 eq.) was added and reaction mixture stirred for 5 - 10 minutes. NaN_3_ (0.72 g, 11 mmol, 2 eq.) was added portion wise and reaction mixture stirred at 80°C overnight. Acetone was evaporated under vaccum. Water was added (50 mL) and the product was extracted with DCM (3 x 50 mL). Combined organic layers were dried over anhydrous MgSO_4_, filtered and evaporated to afford the desired product as a white solid (1.31 g, 88%). ^1^H NMR is in accordance with the literature^4^ .


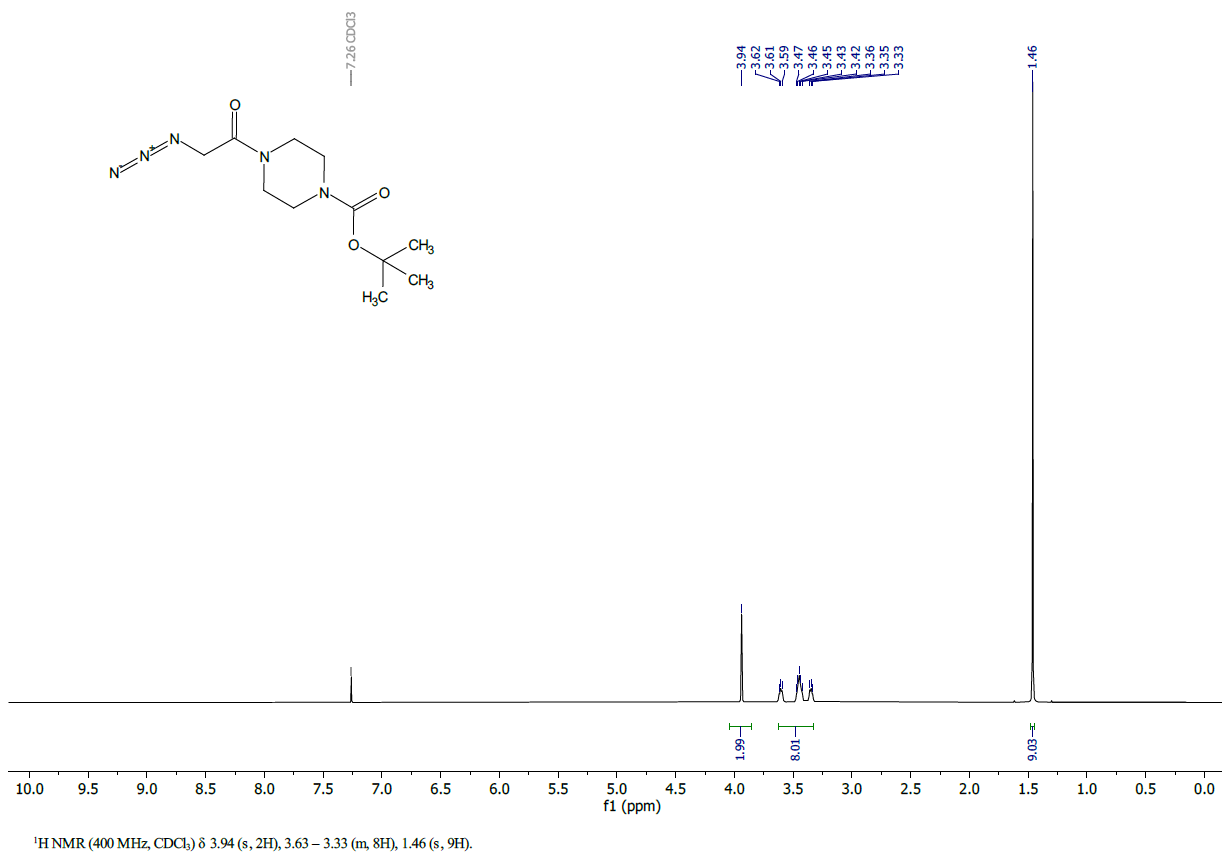


^1^H NMR spectrum of **2**

**3.** To a suspension of 5-formyl salicylic acid (1 g, 6.0 mmol, 1 eq.) and N-hydroxysuccinimide (0.76 g, 6.6 mmol, 1.1 eq.) in THF (10 mL) was added DCC (1.37 g, 6.6 mmol, 1.1 eq.). The mixture was stirred at room temperature for 1 hour and filtered off over a pad of celite. The filtrate was concentrated under reduced pressure then the residue was purified by flash chromatography (heptane/ethyl acetate 7:3 to 3:7) to give **3** (1.1 g, 69 %) as a white solid. ^1^H NMR is in accordance with the literature^5^.


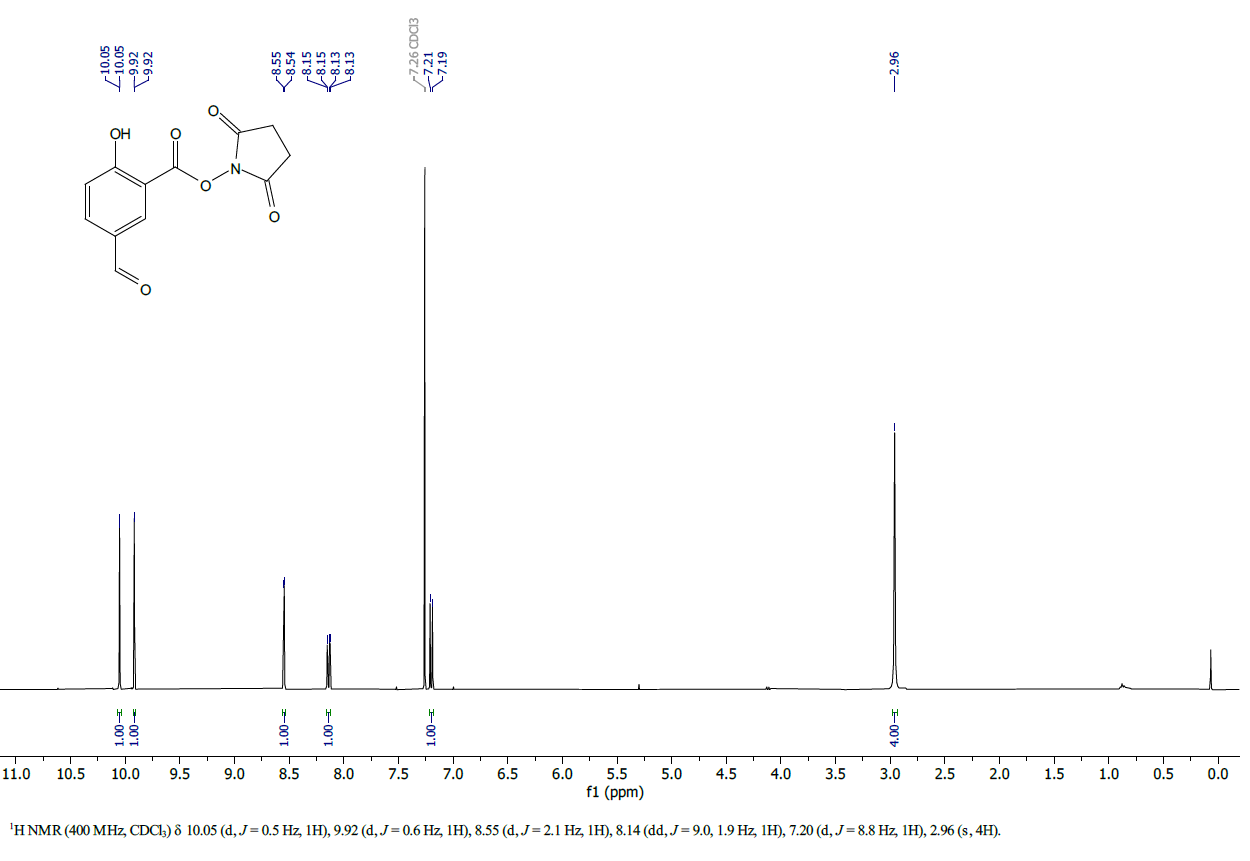


^1^H NMR spectrum of **3**

**4. 2** (0.32 g, 1.2 mmol, 1.2 eq.) was deprotected by dissolving it into a mixture of TFA/DCM (1:1, 5 mL) for 2 hours at room temperature. DCM and TFA were removed *in vacuo*. Deprotected product was added into a solution of **3** (0.26 g, 1.0 mmol, 1 eq.) in 10 mL DMSO, followed by DIPEA (0.5 mL, 3.0 mmol, 3 eq.). The solution was stirred overnight at room temperature. Crude mixture was poured into HCl 1M (10 mL) and extracted with ethyl acetate (3 x 10 mL). The combined organic layers were dried over anhydrous MgSO_4_, filtered and concentrated. The product was purified by flash chromatography (DCM/ethyl acetate 7:3 to 3:7) to give **4** (153 mg, 49%) as a white solid. Rf = 0.3 (DCM/ethyl acetate 4:6).

^1^H NMR (400 MHz, CDCl_3_) δ 9.87 (s, 1H, O=C-H), 7.89 (dd, *J* = 8.5, 2.1 Hz, 1H, H_ar_), 7.83 (d, *J* = 2.1 Hz, 1H, H_ar_), 7.16 (d, *J* = 8.5 Hz, 1H, H_ar_), 4.00 (s, 2H, CH_2_-N_3_), 3.86 – 3.71 (m, 6H, H_piperazine_), 3.56 – 3.49 (m, 2H, H_piperazine_).

^13^C NMR (400 MHz, CDCl_3_+MeOD*-d_4_*) δ 190.59 (O=C-H), 167.98 (O=C-N), 166.41 (O=C-N), 159.73 (C_ar_-OH), 133.00 (C_ar_), 131.25 (C_ar_), 128.80 (C_ar_), 122.76 (C_ar_), 116.58 (C_ar_), 50.68 (C-N_3_), 44.87 (C_piperazine_), 41.93 (C_piperazine_).

HRMS (ESI^+^): *m/z* calculated for C_14_H_15_N_5_O_4_: 317.1124 [M+H]+, found 318.1202.


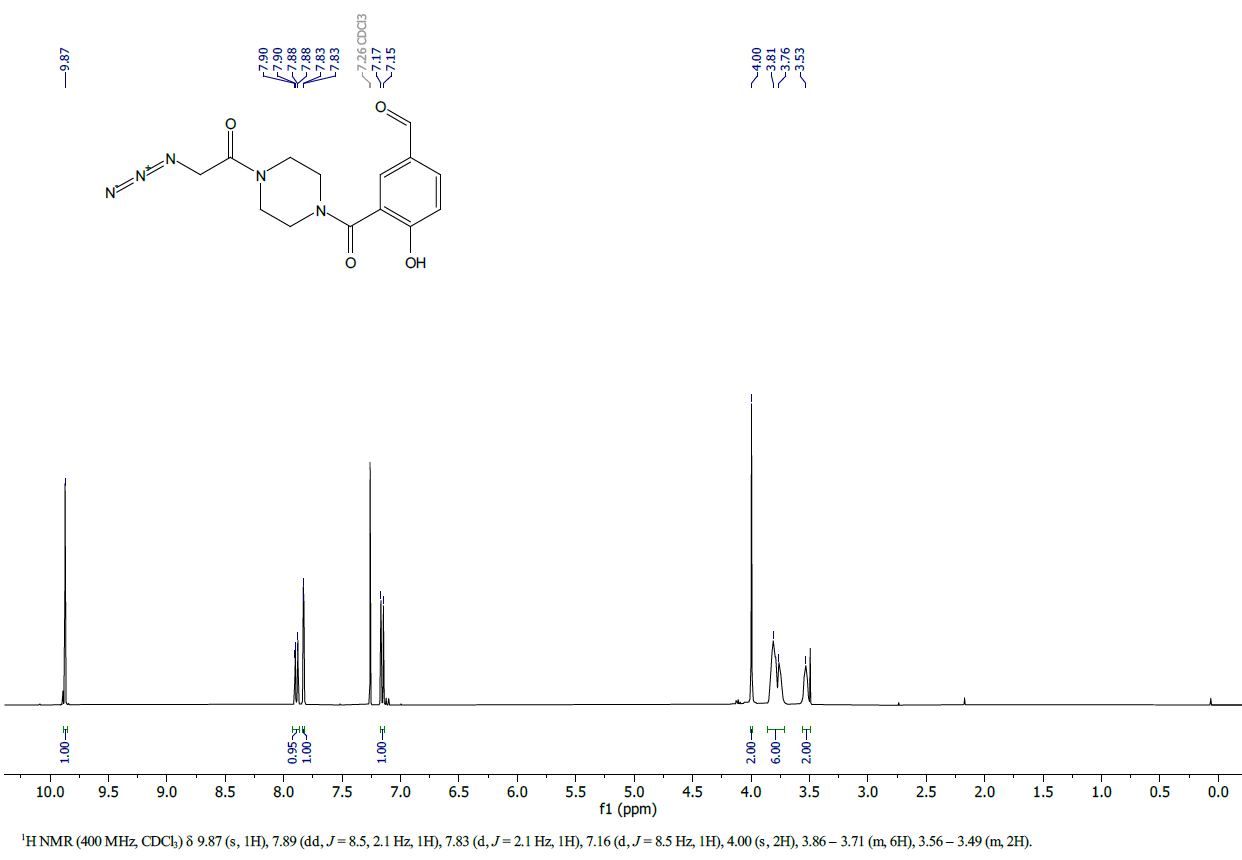


^1^H NMR spectrum of **4**


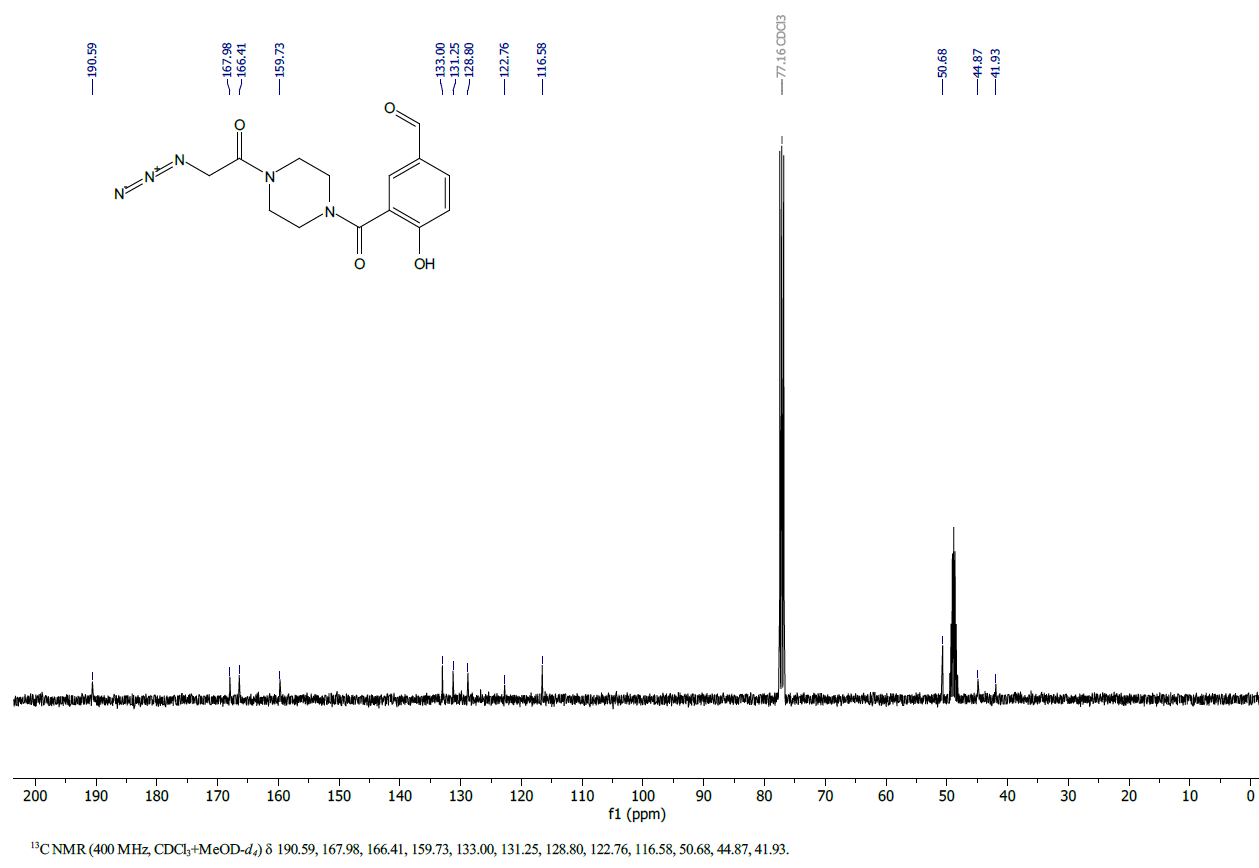


^13^C NMR spectrum of **4**


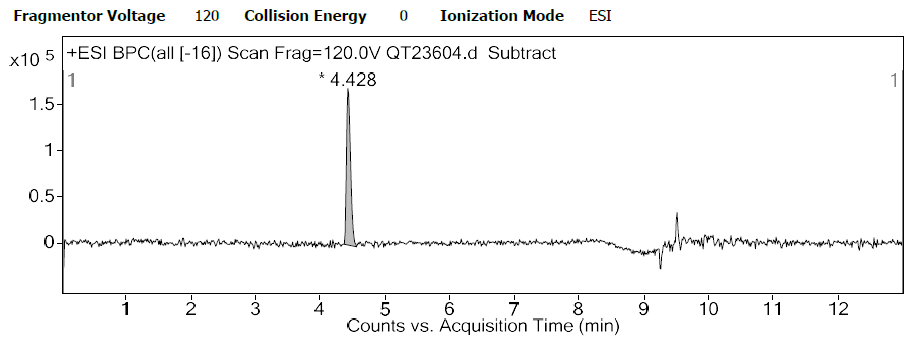


HPLC trace of **4**


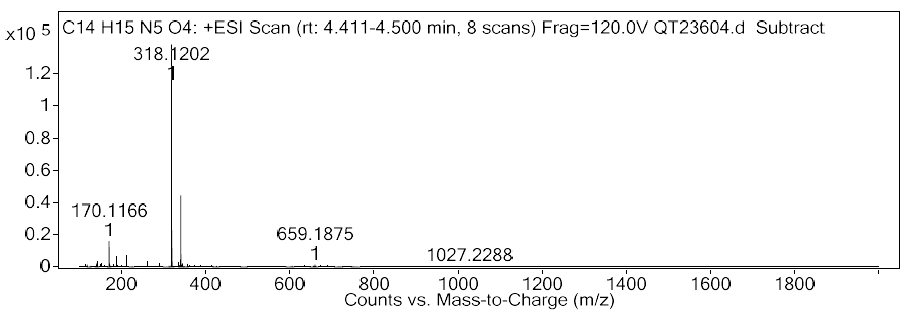


HRMS Spectrum of **4**

**5 (H-Ruby-N_3_). 4** (350 mg, 1.1 mmol, 1 eq.) and 3-diethylaminophenol (401 mg, 2.4 mmol, 2.2 eq.) were dissolved in DCM (5 mL). Triflic acid was added (29 µL, 0.3 mmol, 0.3 eq.), followed by propionic acid (2 mL) to help with solubility. Reaction mixture was stirred in the dark at room temperature for 24 h, until the starting material was completely converted into the aldehyde condensation product. Then chloranil (271 mg, 1.1 mmol, 1 eq.) was added and the reaction mixture was stirred for 24 h in the dark at room temperature. Solvents were evaporated *in vacuo* and the residue was purified by flash chromatography (DCM/MeOH 98:2 to 90:10) to give **5** (117 mg, 16%) as a pink solid. Rf = 0.16 (DCM/MeOH 95:5).

Product was not completely soluble in MeOD-*d4* and was not stable in a CDCl_3_/MeOD-*d4* mixture to obtain a proper NMR analysis.

HRMS (ESI^+^): *m/z* calculated for C_34_H_40_N_7_O_4_: 610.3142 [M]+, found 610.3152.


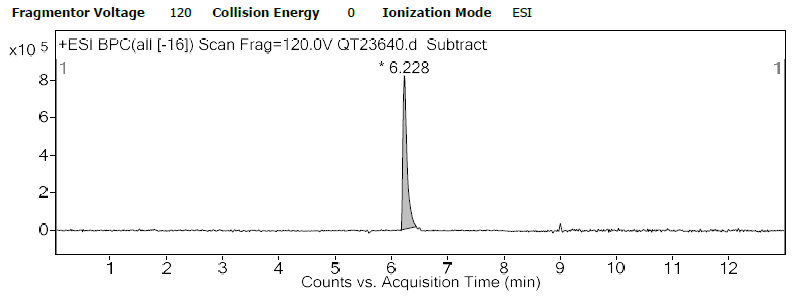


HPLC trace of **5**


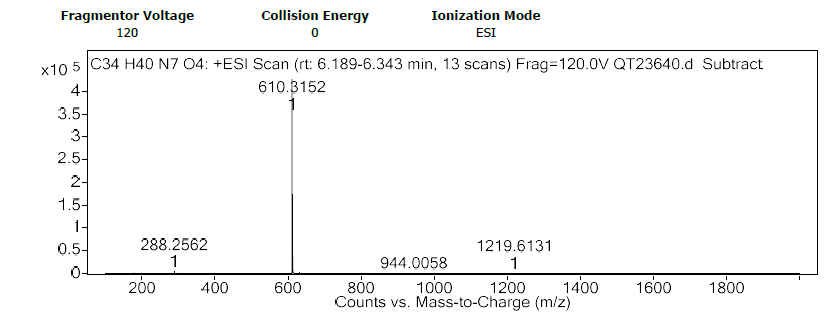


HRMS Spectrum of **5**

**6. Clickable Dextran.** Same procedure as Despras et al. was followed to obtain 220 mg of Clickable 70 kDa Dextran (Degree of Substitution = 70%, Final MW = 104 kDa).^3^

**7. H-Ruby Dextran conjugate.** To a solution of **H-Ruby-N_3_** (2.5 mg, 4.1 µmol) and **clickable dextran** (30 mg, 0.3 µmol) in 400 µL degassed DMF was added copper sulfate pentahydrate (5 mg) and sodium ascorbate (5 mg) in water (100 µL). The reaction was stirred in the dark at 40°C for 30 min. DMF was evaporated and the crude residue was dissolved in aq. 0.1 M EDTA (1 mL) then purified over G-25 size exclusion column and lyophilized to obtain **H-Ruby Dextran conjugate** as a pink solid (28 mg, molar ratio ~ 3 mol dye/mol dextran).

**9. AF488 Dextran conjugate 8.** To a solution of AF488-azide (purchased from Lumiprobe, 2.5 mg, 3.6 µmol) and **clickable dextran** (50 mg, 0.5 µmol) in H_2_O/DMF (4:1, 5 mL) was added copper sulfate pentahydrate (5 mg) and sodium ascorbate (5 mg) in water (100 µL). The reaction was stirred in the dark at 45°C for 4 hours. DMF was evaporated and the crude residue was dissolved in aq. 0.1 M EDTA (1 mL) then purified over G-25 size exclusion column and lyophilized to obtain **AF 488 dextran conjugate** as an orange solid (49 mg, molar ratio ~ 1.7 mol dye/mol dextran).

**Supplementary references:**

1. Kim, S. M., Mizel, D., Qin, Y., Huang, Y. & Schnermann, J. Blood pressure, heart rate and tubuloglomerular feedback in A1AR‐deficient mice with different genetic backgrounds. *Acta Physiol.* 213, 259–267 (2015).

2. Zhao, X. *et al.* Arterial Pressure Monitoring in Mice. *Curr. Protoc. Mouse Biol.* 1, 105–122 (2011).

3. Fang, Y. *et al.* Design and synthesis of tetrahydropyridopyrimidine derivatives as dual GPR119 and DPP-4 modulators. *Bioorg. Chem.* 94, 103390 (2020).

4. Bangalore, P. K. *et al.* Usnic Acid Enaminone-Coupled 1,2,3-Triazoles as Antibacterial and Antitubercular Agents. *J. Nat. Prod.* 83, 26–35 (2020).

5. Despras, G. *et al.* H-Rubies, a new family of red emitting fluorescent pH sensors for living cells. *Chem. Sci.* 6, 5928–5937 (2015).
