# Supplementary material for: Neurovascular coupling and CO2 interrogate distinct vascular regulations
Source: Nat Commun. 2024 Sep 2;15:7635. doi: 10.1038/s41467-024-49698-9 (PMC11369082; doi:10.1038/s41467-024-49698-9)
Supplement: Supplementary file 3 — Description of Additional Supplementary Files [file 41467_2024_49698_MOESM3_ESM.docx]

**Description of Additional Supplementary Files**

**Supplementary Movie 1:** Measurement of GCaMP6 fluorescence in smooth muscle cells (SMCs) during dilation. The two movies show a penetrating arteriole filled with Texas red (red), surrounded by a SMC (GCaMP6, green) and dilated by briefCO2 applied from 10 to 20 s. Left, the movie without motion correction. The arrow indicates the displacement of the SMC outside the ROI (white trace) during dilation. Right, the same movie after applying an autocorrelation algorithm to the left side of the vessel wall. The SMC is stabilized in the ROI and its fluorescence can be measured. The same approach was used to measure GCaMP6 in endothelial cells.

**Supplementary Movie 2:** Large dilation of a pial arteriole (filled with AF488) during prolonged hypercapnic stimulation (10% CO2, 10 min). The perivascular space (PVS, dark) between the arterial wall (green) and the tissue (green, bottom right of the movie) is greatly reduced with dilation. The schematic below illustrates that a shift in the z-plan would increase the PVS, as the initial imaging position in z was set so that the vessel had the largest diameter before stimulation. Note that the autofluorescent basal lamina can be seen as a thin green layer adjacent to the arterial wall.


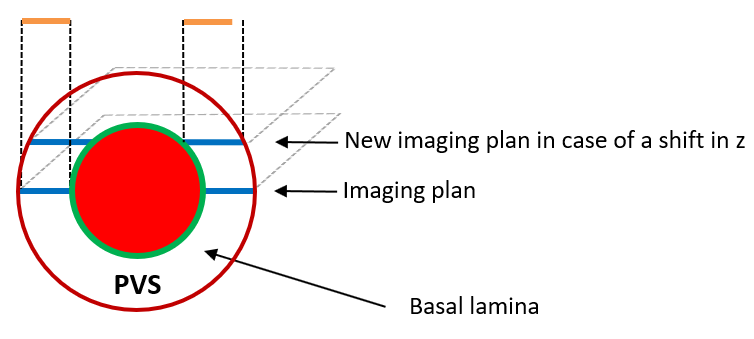


**Supplementary Movie 3:** Another experiment in which autofluorescent spots ensure that dilation is not due to brain movement. The arteriole filled with H-Ruby (pH sensitive dye) dilated after a delay of 6-7 s from the onset of CO2 stimulation, whereas fluorescence increased earlier (within 2-3 s from CO2 onset). Autofluorescent spots in the tissue at 20-30 µm from the vessel wall (top right and bottom center) remain stable, indicating that brain swelling does not occur.
