# Supplementary material for: Neurovascular coupling and CO2 interrogate distinct vascular regulations
Source: Nat Commun. 2024 Sep 2;15:7635. doi: 10.1038/s41467-024-49698-9 (PMC11369082; doi:10.1038/s41467-024-49698-9)
Supplement: Supplementary file 7 — Reporting Summary [file 41467_2024_49698_MOESM7_ESM.pdf]

nature portfolio

Corresponding author(s): Serge Charpak, Marine Tournissac

Last updated by author(s): May 15, 2024

Reporting Summary

Nature Portfolio wishes to improve the reproducibility of the work that we publish. This form provides structure for consistency and transparency in reporting. For further information on Nature Portfolio policies, see our Editorial Policies and the Editorial Policy Checklist.

Statistics

For all statistical analyses, confirm that the following items are present in the figure legend, table legend, main text, or Methods section.

n/a	Confirmed	
☐	☒	The exact sample size (*n*) for each experimental group/condition, given as a discrete number and unit of measurement
☐	☒	A statement on whether measurements were taken from distinct samples or whether the same sample was measured repeatedly
☐	☒	The statistical test(s) used AND whether they are one- or two-sided<br>*Only common tests should be described solely by name; describe more complex techniques in the Methods section.*
☒	☐	A description of all covariates tested
☒	☐	A description of any assumptions or corrections, such as tests of normality and adjustment for multiple comparisons
☐	☒	A full description of the statistical parameters including central tendency (e.g. means) or other basic estimates (e.g. regression coefficient) AND variation (e.g. standard deviation) or associated estimates of uncertainty (e.g. confidence intervals)
☐	☒	For null hypothesis testing, the test statistic (e.g. *F*, *t*, *r*) with confidence intervals, effect sizes, degrees of freedom and *P* value noted<br>*Give P values as exact values whenever suitable.*
☒	☐	For Bayesian analysis, information on the choice of priors and Markov chain Monte Carlo settings
☒	☐	For hierarchical and complex designs, identification of the appropriate level for tests and full reporting of outcomes
☒	☐	Estimates of effect sizes (e.g. Cohen's *d*, Pearson's *r*), indicating how they were calculated

Our web collection on statistics for biologists contains articles on many of the points above.

Software and code

Policy information about availability of computer code

Data collection	Data was collected using custom LabView 2013 software (two photon imaging) and IcoScan from Iconeus (functional ultrasound imaging).
Data analysis	Custom Labview (v2013, v2020), Matlab software (v2018a), GraphPad Prism 6. The codes used for the analyses are available from the corresponding author upon reasonable request. Matlab scripts used for onset determination are available online in the following GitHub repository: https://github.com/laboratory-charpak/NVC_and_CO2

For manuscripts utilizing custom algorithms or software that are central to the research but not yet described in published literature, software must be made available to editors and reviewers. We strongly encourage code deposition in a community repository (e.g. GitHub). See the Nature Portfolio guidelines for submitting code & software for further information.

Data

Policy information about availability of data

All manuscripts must include a data availability statement. This statement should provide the following information, where applicable:

- Accession codes, unique identifiers, or web links for publicly available datasets
- A description of any restrictions on data availability
- For clinical datasets or third party data, please ensure that the statement adheres to our policy

The data generated in the present study are available online in the following GitHub repository: https://github.com/laboratory-charpak/NVC_and_CO2

Research involving human participants, their data, or biological material

Policy information about studies with human participants or human data. See also policy information about sex, gender (identity/presentation), and sexual orientation and race, ethnicity and racism.

Reporting on sex and gender	N/A
Reporting on race, ethnicity, or other socially relevant groupings	N/A
Population characteristics	N/A
Recruitment	N/A
Ethics oversight	N/A

Note that full information on the approval of the study protocol must also be provided in the manuscript.

Field-specific reporting

Please select the one below that is the best fit for your research. If you are not sure, read the appropriate sections before making your selection.

- [x] Life sciences
- [ ] Behavioural & social sciences
- [ ] Ecological, evolutionary & environmental sciences

For a reference copy of the document with all sections, see nature.com/documents/nr-reporting-summary-flat.pdf

Life sciences study design

All studies must disclose on these points even when the disclosure is negative.

Sample size	Sample size was determined related to our previous publications. All our effects were reversible and reproducible for all trials, a phenomenun which is taken into account in statistics but are very important. All statistical tests used in the study were non parametric and the sample number required were fixed by the software GraphPad Prism
Data exclusions	Acquisitions were excluded from the analysis in case of movement artifact due to poor or decreased sedation.
Replication	Several consecutive trials were conducted for each vascular segment in multiple vessels from different mice. Experiments were performed on different set ups, in two laboratories. Similar data were obtained throughout 3 years
Randomization	No randomization was performed.
Blinding	No blinding was performed because the present study did not involved treatments or pathological mouse model.

Reporting for specific materials, systems and methods

We require information from authors about some types of materials, experimental systems and methods used in many studies. Here, indicate whether each material, system or method listed is relevant to your study. If you are not sure if a list item applies to your research, read the appropriate section before selecting a response.

Materials & experimental systems

n/a	Involved in the study	
x		Antibodies
x		Eukaryotic cell lines
x		Palaeontology and archaeology
	x	Animals and other organisms
x		Clinical data
x		Dual use research of concern
x		Plants

Methods

n/a	Involved in the study	
x		ChIP-seq
x		Flow cytometry
x		MRI-based neuroimaging

Animals and other research organisms

Policy information about studies involving animals; ARRIVE guidelines recommended for reporting animal research, and Sex and Gender in Research

Laboratory animals	Adult mice, both males and females, from 3 to 10 months of age were included. The following mouse lines were used and bred in our animal facility: C57/BL6J, NG2-CreERTM,Ai95(RCL-GCaMP6f), Thy1-GCaMP6s (GP4.3), Cdh5-GCaMP8 (#033342, Jackson Laboratory), Cx30-CreERT2 were crossed with Ai95-GCaMP6 mice to generate Cx30-CreERT2;Ai95-GCaMP6 mice, Aldh1l1-Cre/ERT2 (#031008, Jackson Laboratory) were crossed with Ai95-GCaMP6 mice to generate Aldh1l1-CreERT2;Ai95-GCaMP6 mice.
Wild animals	The study did not involved wild animals.
Reporting on sex	Both males and females mice were included. Sex differences were not considered in the present study as sex has not been shown modulate NVC or cerebrovascular reactivity to CO2.
Field-collected samples	The study did not involved samples collected from the field.
Ethics oversight	All animal care and experimentations were performed in accordance with the INSERM Animal Care 396 and Use Committee guidelines and approved by the ethical committee Charles Darwin (comité national 397 de réflexion éthique sur l'expérimentation animale n°5) (protocol number #27135 398 2020091012114621).

Note that full information on the approval of the study protocol must also be provided in the manuscript.

Plants

Seed stocks	N/A
Novel plant genotypes	N/A
Authentication	N/A
